# Pain sensitivity in young adults with juvenile idiopathic arthritis: a quantitative sensory testing study

**DOI:** 10.1186/s13075-020-02345-2

**Published:** 2020-11-05

**Authors:** Ellen Dalen Arnstad, Johanne Marie Iversen, Martin Uglem, Mia Glerup, Pål Richard Romundstad, Trond Sand, Marite Rygg

**Affiliations:** 1 Department of Pediatrics, Levanger Hospital, Nord-Trøndelag Hospital Trust, Levanger, Norway; 2 Department of Clinical and Molecular Medicine, NTNU—Norwegian University of Science and Technology, Trondheim, Norway; 3 Department of Internal Medicine, Nordland Hospital, Bodø, Norway; 4 Department of Neuromedicine and Movement Science, NTNU—Norwegian University of Science and Technology, Trondheim, Norway; 5 Department of Neurology and Clinical Neurophysiology, St. Olavs Hospital, Trondheim, Norway; 6 Department of Pediatrics, Aarhus University Hospital, Aarhus, Denmark; 7 Department of Public Health and Nursing, NTNU—Norwegian University of Science and Technology, Trondheim, Norway; 8 Department of Pediatrics, St. Olavs Hospital, Trondheim, Norway

**Keywords:** Juvenile idiopathic arthritis (JIA), Young adults, Long-term outcomes, Quantitative sensory testing (QST), Pain threshold, Pain perception, Pain sensitivity, Pain sensitization, Self-reported pain

## Abstract

**Background:**

To study for the first-time, pain perception, pain sensitivity, and self-reported pain in young adults with long disease duration of juvenile idiopathic arthritis (JIA) compared with controls.

**Methods:**

Children from Central Norway diagnosed with JIA between 1997 and 2004 were included consecutively in a population-based prospective study. Children with onset 1997–2000 were part of the Nordic JIA cohort. Controls were age- and sex-matched. In 2015–2017, study visits with investigator-blinded quantitative sensory testing (QST) comprising cold and warm detection thresholds (CDT/WDT), cold and heat pain thresholds (CPT/HPT), pressure pain threshold (PPT), and a suprathreshold heat pain test were performed. We constructed separate multilevel models for each variable of detection and pain thresholds with interaction between groups and site adjusted for the effect of age and sex.

**Results:**

Among 96 young adults with JIA, 71% were female, median age was 22.7 years, disease duration was 16.1 years, and 47% had oligoarticular disease. Among 109 controls, 71% were female, and median age was 23.5 years. Participants with JIA had lower pressure pain thresholds (PPTs) (95% CI) compared to controls, upper limb 888 (846,930) versus 1029 (999,1059) kPa and lower limb 702 (670,734) versus 760 (726,794) kPa. Participants with inactive disease had the lowest PPTs and cold pain thresholds (CPTs), compared to those in remission off medication and those with active disease. Minor differences were found regarding CDT/WDT and CPT/HPT in JIA compared to controls. The median (IQR) temperature needed to evoke pain = 6 on a 0–10 numeric rating scale (NRS) in the suprathreshold heat pain tests were lower in JIA than in controls (46 °C (45–47 °C) versus 47 °C (46–48 °C)). We found no associations between self-reported pain and pain thresholds.

**Conclusions:**

Our results indicate for the first time that young adults with long disease duration of JIA may have altered pain perception and sensitivity compared to controls. A clinical implication may be the importance of early treatment to quickly achieve pain-free remission and avoid long-term pain sensitization.

## Background

Juvenile idiopathic arthritis (JIA) is the most common of the pediatric rheumatic diseases, affecting 12.8–23/100,000 children in the Nordic countries [1–3]. JIA is a heterogeneous disease characterized by at least 6 weeks of persistent arthritis of unknown cause with onset before the 16th birthday. JIA is classified into 7 different disease categories [4] with a wide range of disease courses from very mild to severe between categories, and also within each category.

Pain is the most frequent complaint in JIA and has a major negative effect on health-related quality of life (HRQoL), despite modern treatment and good disease control [5–10]. The causes of pain are multifactorial, and both biological and psychosocial factors, such as family relations, level of education, socioeconomic status, and physical health, may contribute to the subjective experience of pain [11, 12].

It has been a concern that neurobiological changes caused by prolonged exposure to pain early in the disease course of JIA, may induce a permanently altered pain perception and sensitization in the central nervous system [13, 14]. The results of such sensitization may be pain elicited by a stimulus that usually does not provoke pain (allodynia) or enhanced pain response to a stimulus that normally provokes minor or moderate pain (hyperalgesia) [15]. The results may be long-term pain persistence or chronic pain in young adults with JIA, irrespective of actual disease activity, and together with reduced pain thresholds and increased response to suprathreshold pain stimuli [16]. To assess pain thresholds as a measure of pain perception and sensitivity, a non-invasive method called quantitative sensory testing (QST) has been used in children and adults both with and without chronic diseases [17–20].

Despite very few studies on pain thresholds in JIA, lower pain thresholds have been demonstrated, both in active and inactive disease [13, 21–23]. In addition, inconsistent results regarding self-reported pain and quantitative sensory testing (QST) results have been reported, with some studies showing correlation between increased self-reported pain and reduced pain thresholds while others have reported no such correlation [13, 21]. Similar results are also found in adults with rheumatoid arthritis (RA) [24, 25]. However, studies on pain thresholds and pain sensitivity in young adults with JIA are lacking. Hence, our study on young adults with JIA may be an important contribution to knowledge of long-term consequences of living with JIA.

The general aim of this study was to examine pain sensitivity and sensory perception among young adults with JIA included in a long-term follow-up study. The primary objective was to study whether pressure pain threshold, cold and heat pain thresholds and suprathreshold pain perception differed compared to matched controls. A secondary objective was to explore potential associations between self-reported pain and pain thresholds.

## Methods

### Patients

Consecutive, newly diagnosed children with juvenile idiopathic arthritis (JIA) from Central Norway, with disease onset between January 1, 1997, and November 30, 2004, were prospectively included in this population-based longitudinal study. Children with onset between January 1, 1997, and June 30, 2000, were part of the 18 years follow-up of the multicenter cohort study, the Nordic JIA study [1]. Children with onset from July 1, 2000, to November 30, 2004, were part of a Norwegian extension of the Nordic JIA study using the same patient enrolment, data collection, and follow-up visits as previously described [1, 26, 27]. To ensure the referral of all eligible patients, all general practitioners and specialists in orthopedics, pediatrics, and rheumatology in the catchment areas repeatedly received letters reminding them of the study. All eligible patients were included and examined at St. Olav Hospital, Trondheim University Hospital. The International League of Associations for Rheumatology (ILAR) classification criteria were used for classification of JIA categories [4].

Participants ≥ 16 years with at least a baseline visit, scheduled to take place 6 months after disease onset, and a follow-up visit between 2015 and 2017 with a quantitative sensory test (QST), were included.

### Data collection

At the baseline visit, demographic and clinical examination, medication, disease activity, and blood tests were registered, and participants or their parents depending of the participant’s age, filled in self-reported questionnaires. After the baseline visit, participants had yearly follow-up visits until they became 18 years and again at the present follow-up. The present study included a visit with clinical examination, blood tests, an updated registration of disease status, and previous and ongoing medication. Self-reported health-related quality of life (HRQoL) questionnaires, including pain measurements, were filled in.

### Controls

From the National Population Register of Norway, age- and sex- matched controls were randomly selected from rural and urban areas of Central Norway. Letters were sent to eligible controls inviting them to participate if they had no rheumatic disease, cancer, or autoimmune diseases. Reminder letters and Short Message Service (SMS) were sent if no answer.

### Measures

Clinical inactive disease was defined according to the American College of Rheumatology (ACR) provisional criteria including; no active joints, no fever, rash, serositis, splenomegaly or generalized lymphadenopathy attributable to JIA, no active uveitis, normal erythrocyte sedimentation rate (ESR) or C-reactive protein (CRP), physician’s global assessment of disease activity with best attainable score indicating no disease activity, and patient-reported morning stiffness ≤ 15 min [28]. According to the preliminary criteria by Wallace, remission on medication was obtained if participants had maintained inactive disease on medication for at least 6 continuous months, and remission off medication if the participants had maintained inactive disease off medication for at least 12 continuous months [29]. Questionnaire on disease-specific self-reported physical disability, was the validated Health Assessment Questionnaire (HAQ), where 0 = no difficulty and 3 = unable to do [30]. As part of HAQ, participants with JIA used a 21-numbered circle visual analog scale (VAS) (0 = no pain, 10 = unbearable pain) to measure self-reported disease-related pain during the last week [31].

To evaluate HRQoL among JIA and controls we used the Medical Outcomes Study 36-Items Short-Form Health Survey (SF-36), a generic multidimensional measurement giving a physical and mental component summary (PCS/MCS) score (range 0–100, 0 = worst, 100 = best) [32]. Based on the general US population’s average score of 50 ± 10, PCS and MCS < 40 were defined as poor health [33]. In addition, two pain questions from SF-36 were used; body pain intensity, scored 1–6 (1 = no pain, 6 = very severe pain), and pain influence on daily activities, scored 1–5 (1 = not at all, 5 = extremely).

Immediately before the experimental test procedure both participants with JIA and controls registered current joint-related pain on a 10-cm VAS scale (0 = no pain, 10 = unbearable pain) and filled in a questionnaire, including questions on medication, food, caffeine, alcohol, and use of tobacco on the day of visit. Female participants registered last menstrual period and hormonal contraceptive use.

### Testing procedure

All tests were performed in one experimental session by the same experienced investigator, who was blinded to whether the participant was a person with JIA or a control. Identical instructions were given by the investigator. The experimental procedure was performed in a constant order and was identical to the protocol used by Iversen et al. [34]. Participants could stop the procedures at any time, by pressing a response button.

### Thermal detection and pain thresholds

To measure thermal thresholds, we used the Somedic MSA thermal stimulator (Somedic SenseLab AB, Norra Mellby, Sweden) with a hand-held Peltier element thermode with contact area of 25 × 50 mm (baseline 32 °C, slope 1 °C/s). Pre-defined cut-off temperatures were 5 and 52 °C. To determine thermal thresholds we used the method of limits [35]. The actual test sequences were always performed in the given order at 2 sites on the body; upper limb (left volar wrist) and lower limb (medial left anterior tibia, 16 cm distal to the inferior patella margin). Participants were instructed to press the response button immediately upon experiencing the first sensations of cold and warmth for detection thresholds (CDT/WDT), and for pain thresholds, whenever they experienced the first sensations of cold and heat pain (CPT/HPT). If the participants pushed the response button by mistake, a new sequence was carried out. To ensure consistency, detection thresholds were measured 5 times, and pain thresholds were measured 4 times [36].

### Pressure pain threshold (PPT)

To estimate the pressure pain threshold (PPT), we used a hand-held digital pressure algometer (Algometer type II, Somedic SenseLab AB, Norra Mellby, Sweden) with a 1-cm^2^ rubber tip fixed to the gauge. Two sites were used; the medial phalanx of the third finger on the left hand, and the right leg, 16 cm distal to the inferior patella margin of the medial anterior tibia. Pressure was applied manually with an even rate perpendicular to the site investigated. Participants were instructed to verbally communicate whenever the sensation of pressure changed into the first sensation of pain, and the application of pressure stopped immediately. PPT was measured 3 times at each site [19, 36].

### Suprathreshold heat pain

To investigate the suprathreshold heat pain response, we used the Somedic MSA thermal stimulator, controlled by the Exposure30 software. Suprathreshold heat pain responses were obtained with continuous stimulation of heat pain on the left volar forearm. The temperature was individually determined corresponding to verbal numeric rating scale (NRS) = 6, ranging from 0 (no pain) to 10 (unbearable pain). At least 2 subsequent 7-s stimuli of heat, with 1-min intervals were used to select the temperature corresponding as close as possible to NRS = 6.

At the actual suprathreshold heat pain procedure, a continuous stimulation of 120 s at the predetermined temperature was performed. Participants were informed to verbally report their NRS pain scores whenever it changed, not being informed that the temperature was held constant. The last NRS value reported every 10 s from 10 to 120 s, were recorded. If the participants terminated the procedure, a new sequence was carried out with 1 °C lower temperature. The suprathreshold heat pain procedure was based on the protocol by Uglem et al. [37], originally based on Granot et al. [38], but with prolonged stimulation time to better reflect alternations in central regulations of pain [39].

### Statistical analyses

To summarize clinical characteristics, we used descriptive statistics with absolute frequencies and percentages for categorial variables, and median with 1st–3rd interquartile range (IQR) or mean with standard deviations (SD) for continuous variables. To investigate group differences in pre-test characteristics, we used Mann-Whitney *U* test or Student’s *t* test. Raw data of cold pain threshold (CPT), heat pain threshold (HPT), and pressure pain threshold (PPT) were presented in median and dot-plots. We constructed separate multilevel models for each variable of detection and pain thresholds with interaction between groups and site adjusted for the effect of age and sex. The models were specified as three-level models with subject as third level random effect and site as second level. Analysis of CPT was done by a multilevel Tobit-model to account for censored values (thresholds below the 5 °C floor temperature). Post hoc contrasts of estimated margins were used for ANOVA-style tests of main, interaction, and simple effects. The results were presented as differences in degrees from baseline (32 °C) in cold and warm detection threshold (CDT/WDT), absolute temperatures in CPT/HPT, and as kilopascal in PPT with 95% CI accompanied by *p* values.

To investigate association of subgroups of JIA with pain thresholds, we constructed additional multilevel models for each variable of the pain thresholds (CPT, HPT, PPT). To evaluate potential associations of self-reported pain with pain threshold values, self-reported pain was included as predictor variable in these models.

To evaluate the model, the residuals were plotted on histograms and quantile-quantile plots and the distributions for pain thresholds were found acceptable. Detection thresholds were transformed logarithmically to optimize the distribution of residuals. A robust estimator of variance was applied for HPT and PPT.

For analyses of the suprathreshold heat pain test, we used a multilevel model with interaction between time and groups, specified as a two-level model with subject as random intercept and time as random slope with an unstructured covariance matrix. Differences in temperature equaling numeric rating scale (NRS) = 6 was explored with Student’s *t* test.

Given 100 participants with JIA and 100 controls, the power was calculated to be 94% to detect a difference corresponding to 0.5 SD and 80% to detect a difference of 0.4 SD in the mean value of a continuous variable between the two groups. For dichotomous variables, the power was calculated close to 80% to detect a difference in proportions between the groups corresponding to 0.5 in JIA and 0.3 in controls.

All statistical analyses were performed using STATA version 16, software (STATA Corp., College Station, TX, USA).

## Results

### Study groups

In the present study 96 of 114 (84%) eligible participants with JIA completed a clinical visit and a quantitative sensory testing (QST). At the study visit, median age of participants with JIA was 22.7 years, 71% were female, and median disease duration was 16.1 years (IQR 14.2–17.1) (Table 1). Clinical characteristics of subgroups of JIA according to disease status are shown in Table 2. In the control cohort median age at visit was 23.5 years, and 71% were female (Table 1).

**Table 1:** Clinical characteristics of the juvenile idiopathic arthritis (JIA) cohort and control group

Characteristics	Juvenile idiopathic arthritis			Control		
	Total no. assessed	Values		Total no. assessed	Values	
Female sex	96	68	(71)	109	77	(71)
Fulltime school or work	94	73	(78)	109	86	(79)
Age at visit, years, median (IQR)	96	22.7	(18.7–26.2)	109	23.5	(20.2–26.7)
Age at disease onset, years, median (IQR)	96	6.4	(2.7–11.7)	–	–	
Disease duration at visit, years, median (IQR)	96	16.1	(14.2–17.1)	–	–	
Oligoarticular JIA at onset	96	51	(53)	–	–	
Oligoarticular JIA at visit^a^	96	45	(47)	–	–	
Not in remission at visit^b^	96	53	(55)	–	–	
DMARDs and/or biologics at visit	96	34	(35)	–	–	
HAQ, median (IQR)	96	0	(0.0–0.125)	–	–	
Women: hormonal contraceptives	68	42	(62)	74	48	(65)
Women: days since LMP, median (IQR)	54	14	(6–26)	69	19	(9–39)
Alcohol consumption at day of visit^c^	95	0	(0)	109	2	(2)
Use of tobacco at day of visit^c^	93	21	(23)	106	22	(21)
Consumption of coffee/tea/cola at day of visit^c^	95	36	(38)	109	28	(26)
Pain or psychotropic medication at day of visit^d^	95	4	(4)	107	2	(2)
SF-36 PCS, mean (± SD)	96	51.1	(± 9.5)	109	55.1	(± 7.9)
SF-36 MCS, mean (± SD)	96	47.7	(± 11.6)	109	47.5	(± 9.7)

Unless otherwise stated, values indicate numbers (%); *no.* numbers; *IQR* interquartile range, 1st–3rd; *DMARDs* disease-modifying anti-rheumatic drugs; *biologics* biologic drugs; *HAQ* Health Assessment Questionnaire, 0–3 (0 = lowest, 3 = highest); *LMP* last menstrual period; *SD* standard deviation; *SF-36* Short-form 36 Health Status Questionnaire, 0–100 (< 40 poor health); *PCS* physical component summary; *MCS* mental component summary

^a^Persistent (no. = 27) and extended (no. = 18) oligoarticular disease

^b^Not in remission off medication for ≥ 12 months according to the definition by Wallace et al.

^c^Consumption after 00.00 on the day of visit

^d^Pain or psychotropic medication after 00.00 on the day of visit, including JIA: ibuprofen (*n* = 1), methylphenidate (*n* = 2), and prednisolone (*n* = 1) and control: paracetamol (*n* = 1) and lisdexamfetamine (*n* = 1)

**Table 2:** Clinical characteristics of subgroups in the juvenile idiopathic arthritis (JIA) cohort

Characteristics	Remission off medication^a^	Inactive disease^a^	Active disease^a^
	Values	Values	Values
	*N* = 43	*N* = 20	*N* = 33
Female sex, *n* (%)	25 (58)	15 (75)	28 (85)
Age at onset, years, median (IQR)	8.3 (3.1–11.8)	3.7 (2.2–8.5)	7.0 (2.6–12.3)
Age at visit, years, median (IQR)	24.2 (20.3–26.5)	18.8 (17.5–21.8)	22.7 (19.2–26.5)
Systemic arthritis, *n* (%)^b^	1 (2)	–	–
Oligoarticular persistent, *n* (%)^b^	17 (40)	5 (25)	5 (15)
Oligoarticular extended, *n* (%)^b^	3 (7)	5 (25)	10 (30)
Polyarthritis RF positive arthritis, *n* (%)^b^	–	1 (5)	1 (3)
Polyarthritis RF negative arthritis, *n* (%)^b^	13 (30)	4 (20)	6 (18)
Psoriatic arthritis, *n* (%)^b^	1 (2)	–	3 (9)
Enthesitis-related arthritis, *n* (%)^b^	3 (7)	2 (10)	2 (6)
Undifferentiated arthritis, *n* (%)^b^	5 (12)	3 (15)	6 (18)
DMARDs ever, *n* (%)	9 (18)	19 (37)	23 (45)
Biologics ever, *n* (%)	2 (5)	13 (35)	22 (60)
HAQ, median (IQR)	0 (0–0)	0 (0–0.125)	0.125 (0–0.25)
SF-36 PCS, mean (± SD)	55.7 (7.0)	52.2 (7.4)	44.6 (9.8)
SF-36 MCS, mean (± SD)	48.8 (12.3)	44.6 (12.7)	48.1 (10.0)

*N* numbers; *IQR* interquartile range, 1st–3rd; *RF* rheumatoid factor; *DMARDs* disease-modifying anti-rheumatic drugs; *Biologics* biologic drugs; *HAQ* Health Assessment Questionnaire, 0–3 (0= lowest, 3 = highest); *SD* standard deviation; *SF-36* Short-form 36 Health Status Questionnaire, 0–100 (< 40 poor health); *PCS* physical component summary; *MCS* mental component summary

^a^Disease status according to the definition by Wallace et al. Remission off medication for ≥ 12 months. Inactive disease on medication for less than 6 months or inactive disease off medication for less than 12 months or remission on medication (inactive disease on medication for more than 6 months). *Active disease* flare or continuous active disease

^b^JIA category according to the International League of Associations for Rheumatology (ILAR) classification criteria

Participants with JIA reported lower mean SF-36 PCS compared to controls (51.1 (± 9.5) versus 55.1 (± 7.9), *p* = 0.004). In the JIA group 16% reported poor health (SF-36 PCS < 40) compared to 7% among controls (*p* = 0.06), and 22% reported poor mental health (SF-36 MCS < 40) compared to 17% among controls (*p* = 0.3). We did not find any group differences in background characteristics regarding alcohol, tobacco, psychotropic, or pain medication on the day of visit.

### Thermal detection thresholds

The results of the detection thresholds are shown in Supplementary Table S1. Thermal detection thresholds were similar between participants with JIA and controls, except that controls detected warmth in the lower limb at a lower temperature change (4.1 °C) than the JIA cohort (4.6 °C).

### Thermal pain thresholds

**Fig. 1:**
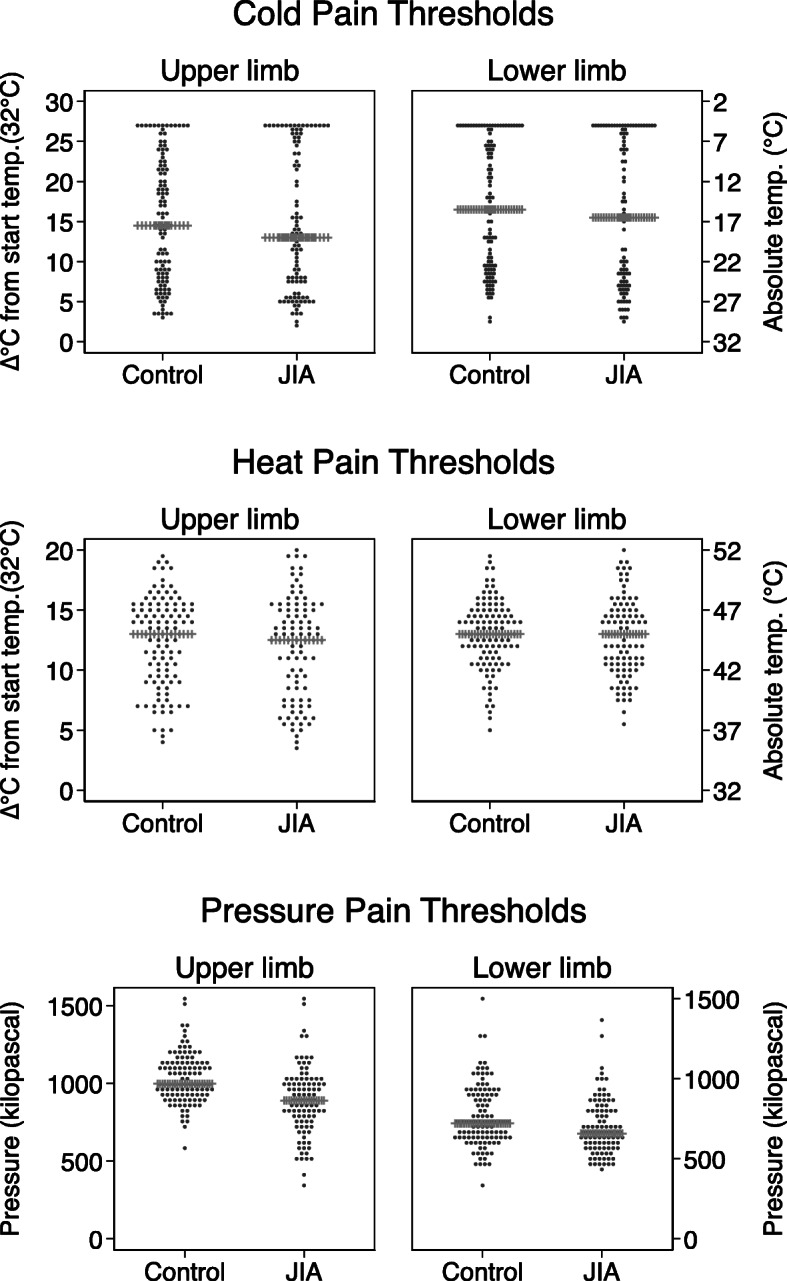
An illustration of the distribution of cold, heat, and pressure pain thresholds (CPT, HPT, and PPT) at upper and lower limb in young adults with juvenile idiopathic arthritis (JIA) and age- and sex-matched controls. The dot-plot illustrating the distribution within each group as dots, and the group median indicated with the horizontal spiked line

Median cold pain threshold (CPT) was slightly lower (higher absolute temperature) in the JIA group compared to controls (Fig. 1 and Supplementary Table S2). Due to the 5 °C floor temperature of the equipment, a substantial number of CPT responses reached this limit and were censored. The number (%) of censored CPT in JIA was 212 (28%) and in controls 198 (23%). The results of CPT and heat pain threshold (HPT) from multilevel models, adjusted for sex and age, are shown in Table 3 with graphically shown estimated margins in Fig. 2. When we subdivided the JIA cohort according to disease status, we found that participants with inactive disease had lower cold pain thresholds (higher absolute temperature), compared to those in remission off medication and to those with an active disease (16.9 °C versus 14.3 °C and 16.2 °C for upper limb, and 17.4 °C versus 11.8 °C and 13.1 °C for lower limb) (Table 3). We did not find any differences for heat pain thresholds.

**Fig. 3:**
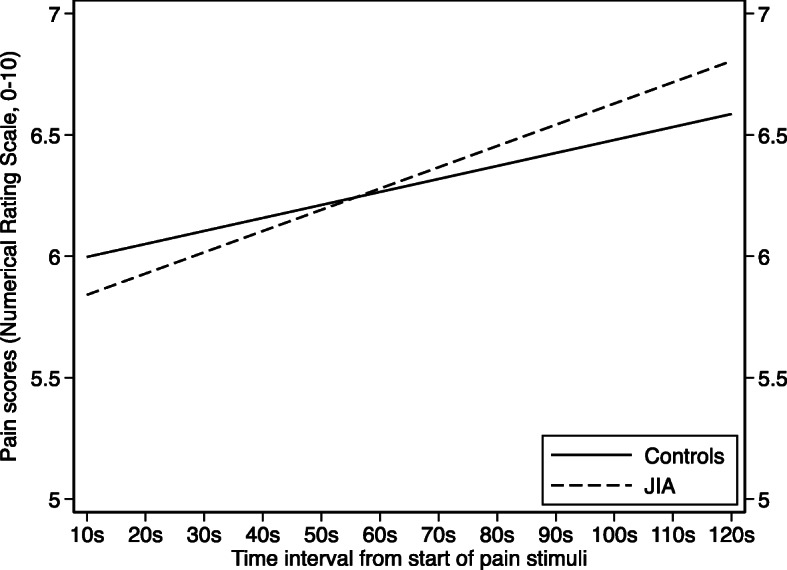
Pain scores during 120-s continuous suprathreshold heat pain stimulation in young adults with juvenile idiopathic arthritis (JIA) and age- and sex-matched controls. Pain scores were measured with numeric rating scale (NRS), range 0–10 (0 = no pain, 10 = unbearable pain). The estimated margins of the multilevel model are graphically illustrated with time on the *x*-axis and NRS scores on the *y*-axis. In both groups, the NRS scores increased during the 120-s stimulation, but it was a trend towards steeper increase in the JIA group

**Table 3:** Thermal pain thresholds and pressure pain thresholds among juvenile idiopathic arthritis (JIA) and controls^*^

	*n*	Cold pain threshold^**a**^		Heat pain threshold		Pressure pain threshold	
		Absolute temperature °C (95% CI)		Absolute temperature °C (95% CI)		Kilopascal (95% CI)	
		Upper limb	Lower limb	Upper limb	Lower limb	Upper limb	Lower limb
Control	109	15.9 (13.8, 18.0)	13.2 (11.1, 15.3)	44.3 (43.6, 45.0)	44.9 (44.3, 45.4)	1029 (999, 1059)	760 (726, 794)
JIA (total group)^b^	96	15.5 (13.1, 17.8)	13.4 (11.1, 15.8)	44.0 (43.1, 44.8)	44.8 (44.2, 45.4)	888 (846, 930)	702 (670, 734)
		*p* = 0.8	*p* = 0.9	*p* = 0.5	*p* = 0.8	*p* < 0.001	*p* = 0.01
JIA							
Remission off med.^c^	43	14.3 (10.7, 17.8)	11.8 (8.2, 15.4)	43.6 (42.2, 45.0)	44.8 (43.8, 45.8)	893 (823, 962)	731 (674, 788)
		*p* = 0.4	*p* = 0.5	*p* = 0.4	*p* = 0.9	*p* < 0.001	*p* = 0.4
Inactive disease^c^	20	16.9 (11.9, 22.0)	17.4 (12.4, 22.4)	44.2 (42.4, 46.0)	44.9 (43.5, 46.3)	836 (761, 910)	626 (574, 677)
		*p* = 0.7	*p* = 0.1	*p* = 0.9	*p* = 1.0	*p* < 0.001	*p* < 0.001
Active disease^c^	33	16.2 (12.4, 20.0)	13.1 (9.2, 17.0)	44.3 (43.1, 45.4)	44.6 (43.9, 45.3)	910 (842, 978)	707 (662, 752)
		*p* = 0.9	*p* = 1.0	*p* = 0.9	*p* = 0.6	*p* = 0.002	*p* = 0.06

^*^Multilevel modeling with predicted values as absolute temperature with 95% CI, adjusted for age and sex

*CI* confidence interval, *p p* value comparing controls with JIA disease group (either total group or according to disease status)

^a^A substantial number were censored because the participants did not reach their cold pain thresholds due to floor temperature of the hardware was 5 °C; 212 (28%) in the JIA group and 198 (23%) in the control group. We accounted for these censored responses in the statistical analyses

^b^Separate multilevel model of the total JIA cohort

^c^Disease status according to the definition by Wallace et al. *Remission off med.* remission off medication for ≥ 12 months, *Inactive disease* inactive disease on medication for less than 6 months or inactive disease off medication for less than 12 months or remission on medication (inactive disease on medication for more than 6 months). *Active disease* flare or continuous active disease

The upper limb was more sensitive to thermal stimuli than the lower limb for both JIA and controls, except for CPT in individuals with inactive JIA. Females and younger participants were more sensitive to thermal stimuli compared to males and older participants. Only minor differences between JIA categories were found, but the numbers in some categories were small (Table 2).

### Pressure pain thresholds (PPTs)

**Fig. 2:**
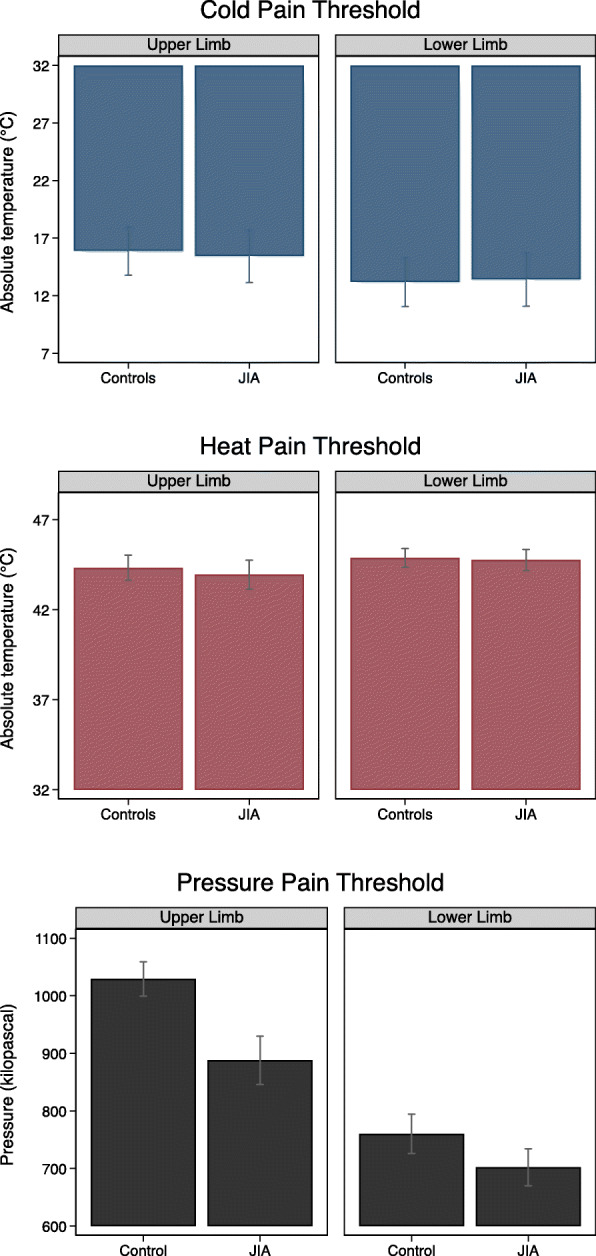
Graphic illustration of cold, heat, and pressure pain thresholds (CPT, HPT, and PPT) in JIA and controls as estimated margins with 95% CI from the multilevel models, adjusted for age and sex. The *y*-axis represents absolute temperature (°C) in CPT and HPT and kilopascal (kPa) in PPT. Since the censored CPT values are accounted for in the estimated margins of CPT, these results are not directly comparable with the results in Fig. 1 and Supplementary Table S2

Median pressure pain threshold (PPT) was lower among participants with JIA compared to controls both at upper and lower limb (Fig. 1 and Supplementary Table S2). Results from the multilevel regression model, adjusted for sex and age, with estimated margins (95% CI) confirmed these findings, 888 (846, 930) versus 1029 (999, 1059) kPa for upper limb (*p* < 0.001) and 702 (670, 734) versus 760 (726, 794) kPa for lower limb (*p* = 0.01) (Table 3), with graphically shown estimated margins in Fig. 2. In the JIA cohort, participants with inactive disease had lower PPT compared to both individuals in remission off medication and to those with active disease. The results were similar for both upper and lower limbs.

### Suprathreshold heat pain

The median (IQR 1st–3rd) temperature equaling numeric rating scale (NRS) = 6, differed between individuals with JIA and controls (46 °C (45–47 °C) versus 47 °C (46–48 °C), *p* = 0.02). During the continuous stimulation of heat pain for 120 s, NRS increased from 5.84 to 6.80 in the JIA group and from 6.00 to 6.59 in the control group (Fig. 3), giving a gradient (95% CI) of 0.96 (0.61, 1.32) in individuals with JIA and 0.59 (0.25, 0.92) in controls (*p* = 0.1). Two participants (one from each group) did not obtain a complete test and were excluded from the analyses.

### Self-reported pain

Participants with JIA reported higher current joint-related VAS pain on the day of visit compared to controls, mean (± SD) VAS pain 1.0 (± 1.7) versus 0.2 (± 1.0), *p* < 0.001, respectively (Table 4). A similar trend was seen for pain intensity and pain interference measured with SF-36. Regardless of which measurement used for self-reported pain, participants with active disease reported the highest pain scores. On the contrary, participants in remission off medication reported pain scores more similar to controls.

**Table 4:** Self-reported pain among participants with juvenile idiopathic arthritis (JIA) and controls

	*n*	Pain intensity^a^	Pain interference^b^	VAS current joint-related pain^c^	VAS disease-related pain^d^
Control	109	2.2 (± 1.3)	1.4 (± 0.7)	0.2 (± 1.0)	–
JIA (total group)	96	2.5 (± 1.3)	1.7 (± 1.0)	1.0 (± 1.7)	1.7 (± 2.2)
JIA					
Remission off med.^e^	43	1.9 (± 1.1)	1.5 (± 1.0)	0.5 (± 1.2)	0.4 (± 1.2)
Inactive disease^e^	20	2.7 (± 1.3)	1.5 (± 0.9)	0.9 (± 1.7)	1.9 (± 2.0)
Active disease^e^	33	3.3 (± 1.2)	2.1 (± 0.9)	1.8 (± 1.9)	3.2 (± 2.5)

Values are mean (± SD) unless otherwise indicated; *n* numbers, *VAS* visual analog scale

^a^Self-reported body pain intensity during the last 4 weeks, measured with SF-36 questionnaire, 1–6 (1 = no pain, 6 = very severe pain)

^b^Self-reported pain influence on daily activities during the last 4 weeks, measured with SF-36 questionnaire, 1–5 (1 = not at all, 5 = extremely)

^c^Self-reported current joint-related pain the day of visit, measured with 10 cm continuous VAS (0 = no pain, 10 = unbearable pain). One of 33 participants with active disease did not fill in the VAS current joint-related pain

^d^Self-reported disease-related pain last week measured with 21 numbered 0–10 VAS (0 = no pain, 10 = unbearable pain)

^e^Disease status according to the definition by Wallace et al. *Remission off med.* remission off medication for ≥ 12 months. *Inactive disease* inactive disease on medication for less than 6 months or inactive disease off medication for less than 12 months or remission on medication (inactive disease on medication for more than 6 months). *Active disease* flare or continuous active disease

We did not find any association between self-reported pain scores, and the quantitative sensory testing (QST) results, neither among the JIA group, nor among controls (Supplementary Tables S3 and S4).

## Discussion

In our population-based JIA cohort, we found negligible differences in detection thresholds and heat pain threshold (HPT), and slightly lower median cold pain threshold (CPT) (higher absolute temperature) compared to controls. Participants with JIA had lower pressure pain threshold (PPT) compared to controls, and participants with inactive JIA had the lowest thresholds. Results from the suprathreshold heat pain test showed that participants with JIA reported lower temperature equaling numeric rating scale (NRS) = 6 compared to controls and tended to have a steeper rise in the NRS score. Self-reported pain scores were higher in the JIA cohort compared to controls, and participants with active JIA had the highest pain scores. No associations between self-reported pain and pain thresholds were found.

The main strengths of our study are the population-based design and long and equal follow-up time for all JIA participants, which enhances the generalizability of the findings and allows for evaluation after long disease duration, even though the quantitative sensory testing (QST) results are cross-sectional. Another strength is the standardized experimental procedure using one single experienced investigator blinded to group adherence. Comparison to age- and sex-matched randomly drawn controls adds further strength to the study. Some limitations must be mentioned. Since the participants are mostly Caucasians, the generalizability to other ethnic groups may be questioned. As the study had a population-based design and the population in Central Norway is almost exclusively Caucasians, we could not include more ethnic groups. The floor temperature of 5 °C resulted in a substantial number of censored cold pain threshold (CPT) responses. However, there were no group differences in the frequency of censored values, and we included statistical analyses accounting for censored responses. Due to the criteria for inactive disease and remission, internal subgroups of JIA only reflect disease status during the last year. Further, a challenge with all self-reported disease-related pain reports is whether the pain experienced by the participants are related and proportionated to their disease or might be influenced by other factors as, e.g., relationship with family and peers, level of education, physical activity, musculoskeletal co-conditions, and cultural or psychosocial factors. Different access to health care services may also influence on pain experience. In Norway, the health care system is mostly governmental and free of charge, so we do not think this will influence our results but may influence the generalizability to other countries with different health care systems than ours. Due to lack of information on socioeconomic status, the impact of this was not included in the analysis. Neurological diseases were not among the exclusion criteria, which could be a limitation. However, both participants with JIA and controls were asked if they had other diseases, but not specifically asked about neurological conditions.

To our knowledge, this is the first study performing quantitative sensory testing (QST) in young adults with JIA. A few studies have been done in children and adolescents (6–17 years) with JIA with comparison to controls [13, 21–23]. Consistent with our study, all these studies found lower pressure pain threshold (PPT) in the JIA group compared to controls. Only one of these studies included cold pain threshold (CPT) and heat pain threshold (HPT), in addition to PPT [22]. Their findings that JIA had consistently lower pain thresholds than controls in all modalities are different from our results. Possible explanations may be differences in methods, the control groups, and the JIA cohort. Since they used historical control groups from the USA and Europe there are some discrepancies between the modalities, equipment, test sites, and also lack of age- and sex-matching with the JIA group. This is in contrast to our study where JIA and age- and sex-matched control groups performed identical procedures. While we included the whole disease spectrum of JIA, which is a heterogeneous disease group, their participants were skewed into the more severe end. Two of these studies included a broader spectrum of JIA categories and were therefore more comparable to our study cohort [13, 21].

Another important issue regarding different responses to different test modalities is that pressure pain threshold (PPT) is the only quantitative sensory testing (QST) which reflects deep somatic pain corresponding to pain in JIA, in contrast to cold pain threshold (CPT) and heat pain threshold (HPT) which reflect superficial pain sensitivity [40, 41].

In addition to QST studies, a Danish group investigating reaction to cold pressor pain has suggested reduced pain tolerance among children with JIA [42, 43]. Due to their study design, these studies cannot be directly compared with ours.

Held together, all these studies suggest an altered pain sensitivity among JIA compared to controls. Our results mainly support this theory and furthermore expand the follow-up period into adult life.

In adults with rheumatoid arthritis (RA), there are several studies including quantitative sensory testing (QST), and some with comparison to healthy controls [24, 25, 44–49]. Some studies have included selected groups of RA or only females, with considerably older age groups than our study. Most studies have performed pressure pain threshold (PPT) with some differences in procedures and site selections. Although there are methodological differences, the results on PPT are in accordance with our results, strengthening the suggestion of altered sensitivity to pain also in adults with arthritis.

Studies on associations between disease activity and quantitative sensory testing (QST) have shown inconsistent results. Hogeweg et al. found that individuals with active disease had lower pressure pain threshold (PPT) than those with inactive disease, but Leegaard et al. and Cornelissen et al. did not find this correlation [13, 21, 22]. In our study we found that participants with active disease had lower cold pain threshold (CPT) (higher absolute temperature), but small differences in PPT compared to participants in remission off medication. Since their inactive disease groups may also include those in remission off medication, in contrast to our study, this could have affected the results. However, even more interesting was that our participants with inactive JIA had consistently lower CPT and PPT compared to both participants in remission off medication and those with active disease. A possible explanation could be that individuals in long-term remission off medication, due to their mild disease, may never have experienced altered pain sensitivity and are more comparable to controls. On the other hand, we could not rule out that alternations in pain processing pathways are mild and reversible in mild cases of JIA. A longitudinal study with repeated assessments over several years might answer this question more definitely. In contrast, participants with more severe disease courses whether inactive or active at the 16-year visit, have probably experienced long-term disease activity as suggested by the higher proportions of medication used during the disease course, and may have permanently or at least extendedly altered pain sensitivity. The ongoing joint-related pain experienced by participants with active JIA at the day of testing may have distracted the participants from the experimental pain. Moreover, we cannot rule out that some internal group differences in the JIA cohort might have influenced the results, although probably to a small extent because the differences were inconsistent, and the analyses adjusted for sex and age.

Increased suprathreshold pain response has been suggested as a sign of altered central sensitization [38]. In our study, both the JIA and the control groups had an increase in numeric rating scale (NRS) scores during the suprathreshold procedure. In the JIA group, a trend towards steeper increase compared to controls indicated sensitization, but the difference was small and should be interpreted with caution. The lower temperature needed to provoke pain equaling NRS = 6 supports the hypothesis of hyperalgesia in the JIA group. To our knowledge, no directly comparable studies on prolonged suprathreshold heat pain in JIA exist. However, the lower pain tolerance measured by cold pressor pain test in JIA, as well as increased temporal summation investigated in RA, supports our results [25, 42].

In accordance with our results, several studies show higher self-reported pain among individuals with JIA compared to controls [21, 50, 51]. We did not find any association between self-reported pain and pain thresholds. This is in accordance with Leegaard et al., but opposite to Hogeweg et al. [13, 21]. In RA studies, the results are also inconsistent [24, 25, 44]. While quantitative sensory testing (QST) mirrors alternations in the sensory system, self-reported pain questionnaires evaluate subjective pain intensity and interference [41, 52]. This difference might to some extent account for the lack of congruence between these tools.

## Conclusions

In conclusion, we found lower pressure pain threshold (PPT) and lower temperature equaling numeric rating scale (NRS) = 6 in young adults with JIA, compared to controls. Individuals with inactive JIA had the lowest PPT and cold pain threshold (CPT). No associations between self-reported pain and pain thresholds were found. This is the first study using quantitative sensory testing (QST) in young adults with JIA. Taken together, the study indicates for the first-time, altered pain perception and sensitization after long disease duration of JIA. A clinical implication of our study may be to emphasize the importance of early intensive treatment to quickly achieve pain-free remission and avoid long-term pain sensitization.

## Supplementary information

**Additional file 1: Supplementary Table S1.** Thermal detection thresholds among juvenile idiopathic arthritis (JIA) and controls.

**Additional file 2: Supplementary Table S2.** Median thermal and pressure pain thresholds in juvenile idiopathic arthritis (JIA) and controls.

**Additional file 3: Supplementary Table S3.** Estimated differences in pain threshold per unit score in self-reported VAS pain.

**Additional file 4: Supplementary Table S4.** Estimated differences in pain threshold: mild versus severe pain intensity / low versus high pain interference.

## Data Availability

The datasets generated and/or analyzed during the current study are not publicly available for ethical reasons, as well as privacy reasons but are available from the corresponding author on reasonable request.

## Abbreviations

ACR: The American College of Rheumatology
CDT: Cold detection threshold
CI: Confidence interval
CPT: Cold pain threshold
CRP: C-reactive protein
ESR: Erythrocyte sedimentation rate
HAQ: Health Assessment Questionnaire
HPT: Heat pain threshold
HRQoL: Health-related quality of life
ILAR: The International League of Associations for Rheumatology
IQR: Interquartile range, 1st -3rd
JIA: Juvenile idiopathic arthritis
NRS: Numeric rating scale
PPT: Pressure pain threshold
QST: Quantitative sensory testing
RA: Rheumatoid arthritis
SD: Standard deviation
SF-36 MCS/PCS: The Medical Outcomes Study 36-Items Short-Form Health Survey, mental/physical component summary
VAS: Visual analog scale
WDT: Warm detection threshold

## References

[1] Berntson L, Andersson Gare B, Fasth A, Herlin T, Kristinsson J, Lahdenne P (2003). Incidence of juvenile idiopathic arthritis in the Nordic countries. A population based study with special reference to the validity of the ILAR and EULAR criteria. J Rheumatol.

[2] Riise OR, Handeland KS, Cvancarova M, Wathne KO, Nakstad B, Abrahamsen TG (2008). Incidence and characteristics of arthritis in Norwegian children: a population-based study. Pediatrics..

[3] Berthold E, Mansson B, Kahn R (2019). Outcome in juvenile idiopathic arthritis: a population-based study from Sweden. Arthritis Res Ther.

[4] Petty RE, Southwood TR, Manners P, Baum J, Glass DN, Goldenberg J (2004). International League of Associations for Rheumatology classification of juvenile idiopathic arthritis: second revision, Edmonton, 2001. J Rheumatol.

[5] Gutierrez-Suarez R, Pistorio A, Cespedes Cruz A, Norambuena X, Flato B, Rumba I (2007). Health-related quality of life of patients with juvenile idiopathic arthritis coming from 3 different geographic areas. The PRINTO multinational quality of life cohort study. Rheumatology (Oxford).

[6] Klotsche J, Minden K, Thon A, Ganser G, Urban A, Horneff G (2014). Improvement in health-related quality of life for children with juvenile idiopathic arthritis after start of treatment with etanercept. Arthritis Care Res (Hoboken).

[7] Tollisen A, Selvaag AM, Aulie HA, Lilleby V, Aasland A, Lerdal A (2018). Physical functioning, pain, and health-related quality of life in adults with juvenile idiopathic arthritis: a longitudinal 30-year followup study. Arthritis Care Res (Hoboken).

[8] Bromberg MH, Connelly M, Anthony KK, Gil KM, Schanberg LE (2014). Self-reported pain and disease symptoms persist in juvenile idiopathic arthritis despite treatment advances: an electronic diary study. Arthritis Rheumatol.

[9] Lomholt JJ, Thastum M, Herlin T (2013). Pain experience in children with juvenile idiopathic arthritis treated with anti-TNF agents compared to non-biologic standard treatment. Pediatr Rheumatol Online J.

[10] Wipff J, Sparsa L, Lohse A, Quartier P, Kahan A, Deslandre CJ (2016). Impact of juvenile idiopathic arthritis on quality of life during transition period at the era of biotherapies. Joint Bone Spine.

[11] Stinson JN, Luca NJ, Jibb LA (2012). Assessment and management of pain in juvenile idiopathic arthritis. Pain Res Manag.

[12] Weiss JE, Luca NJ, Boneparth A, Stinson J (2014). Assessment and management of pain in juvenile idiopathic arthritis. Paediatr Drugs.

[13] Hogeweg JA, Kuis W, Huygen AC, de Jong-de vos van Steenwijk C, Bernards AT, Oostendorp RA, et al. The pain threshold in juvenile chronic arthritis. Br J Rheumatol 1995;34(1):61–67. 10.1093/rheumatology/34.1.61 7881842

[14] Latremoliere A, Woolf CJ (2009). Central sensitization: a generator of pain hypersensitivity by central neural plasticity. J Pain.

[15] Flor H, Turk DC (2011). Chronic pain: an integrated biobehavioral approach.

[16] La Hausse de Lalouviere L, Ioannou Y, Fitzgerald M (2014). Neural mechanisms underlying the pain of juvenile idiopathic arthritis. Nat Rev Rheumatol.

[17] Blankenburg M, Boekens H, Hechler T, Maier C, Krumova E, Scherens A (2010). Reference values for quantitative sensory testing in children and adolescents: developmental and gender differences of somatosensory perception. Pain..

[18] Pas R, Ickmans K, Van Oosterwijck S, Van der Cruyssen K, Foubert A, Leysen L (2018). Hyperexcitability of the central nervous system in children with chronic pain: a systematic review. Pain Med.

[19] Rolke R, Baron R, Maier C, Tölle TR, Treede RD, Beyer A (2006). Quantitative sensory testing in the German Research Network on Neuropathic Pain (DFNS): standardized protocol and reference values. Pain.

[20] Engstrøm M, Hagen K, Bjørk MH, Stovner LJ, Gravdahl GB, Stjern M (2013). Sleep quality, arousal and pain thresholds in migraineurs: a blinded controlled polysomnographic study. J Headache Pain.

[21] Leegaard A, Lomholt JJ, Thastum M, Herlin T (2013). Decreased pain threshold in juvenile idiopathic arthritis: a cross-sectional study. J Rheumatol.

[22] Cornelissen L, Donado C, Kim J, Chiel L, Zurakowski D, Logan DE (2014). Pain hypersensitivity in juvenile idiopathic arthritis: a quantitative sensory testing study. Pediatr Rheumatol Online J.

[23] Hogeweg JA, Kuis W, Oostendorp RA, Helders PJ (1995). General and segmental reduced pain thresholds in juvenile chronic arthritis. Pain.

[24] Bagnato G, De Andres I, Sorbara S, Verduci E, Corallo G, Ferrera A (2015). Pain threshold and intensity in rheumatic patients: correlations with the Hamilton Depression Rating scale. Clin Rheumatol.

[25] Heisler AC, Song J, Dunlop DD, Wohlfahrt A, Bingham CO, Bolster MB (2020). Association of pain centralization and patient-reported pain in active rheumatoid arthritis. Arthritis Care Res (Hoboken)..

[26] Nordal E, Zak M, Aalto K, Berntson L, Fasth A, Herlin T (2011). Ongoing disease activity and changing categories in a long-term nordic cohort study of juvenile idiopathic arthritis. Arthritis Rheum.

[27] Glerup M, Rypdal V, Arnstad ED, Ekelund M, Peltoniemi S, Aalto K (2020). Long-term outcomes in juvenile idiopathic arthritis: eighteen years of follow-up in the population-based Nordic juvenile idiopathic arthritis cohort. Arthritis Care Res (Hoboken).

[28] Wallace CA, Giannini EH, Huang B, Itert L, Ruperto N (2011). American College of Rheumatology provisional criteria for defining clinical inactive disease in select categories of juvenile idiopathic arthritis. Arthritis Care Res (Hoboken).

[29] Wallace CA, Ruperto N, Giannini E; Childhood Arthritis and Rheumatology Research Alliance; Pediatric Rheumatology International Trials Organization; Pediatric Rheumatology Collaborative Study Group. Preliminary criteria for clinical remission for select categories of juvenile idiopathic arthritis. J Rheumatol. 2004;31(11):2290–4. 15517647

[30] Fries JF, Spitz P, Kraines RG, Holman HR (1980). Measurement of patient outcome in arthritis. Arthritis Rheum.

[31] Filocamo G, Davi S, Pistorio A, Bertamino M, Ruperto N, Lattanzi B (2010). Evaluation of 21-numbered circle and 10-centimeter horizontal line visual analog scales for physician and parent subjective ratings in juvenile idiopathic arthritis. J Rheumatol.

[32] Ware JE, Jr. MOT, Health Assessment Lab, Quality Metric Incorporated : SF-36^( !R) Health Survey^<(C)> 1988, 2002. IQOL SF-36 Standard German (Austria). 2002.

[33] Ware JE (2000). SF-36 health survey update. Spine (Phila Pa 1976).

[34] Iversen JM, Uglem M, Indredavik MS, Romundstad PR, Nilsen KB, Sand T (2018). Pain sensitivity and thermal detection thresholds in young adults born preterm with very low birth weight or small for gestational age at term compared with controls. J Pain.

[35] Chong PS, Cros DP (2004). Technology literature review: quantitative sensory testing. Muscle Nerve.

[36] Backonja MM, Attal N, Baron R, Bouhassira D, Drangholt M, Dyck PJ (2013). Value of quantitative sensory testing in neurological and pain disorders: NeuPSIG consensus. Pain.

[37] Uglem M, Omland PM, Nilsen KB, Tronvik E, Stovner LJ, Hagen K (2017). Does pain sensitivity change by migraine phase? A blinded longitudinal study. Cephalalgia.

[38] Granot M, Granovsky Y, Sprecher E, Nir RR, Yarnitsky D (2006). Contact heat-evoked temporal summation: tonic versus repetitive-phasic stimulation. Pain..

[39] Suzan E, Aviram J, Treister R, Eisenberg E, Pud D (2015). Individually based measurement of temporal summation evoked by a noxious tonic heat paradigm. J Pain Res.

[40] Treede RD, Rolke R, Andrews K, Magerl W (2002). Pain elicited by blunt pressure: neurobiological basis and clinical relevance. Pain..

[41] Gierthmühlen J, Schneider U, Seemann M, Freitag-Wolf S, Maihöfner C, Enax-Krumova EK (2019). Can self-reported pain characteristics and bedside test be used for the assessment of pain mechanisms? An analysis of results of neuropathic pain questionnaires and quantitative sensory testing. Pain..

[42] Thastum M, Zachariae R, Herlin T (2001). Pain experience and pain coping strategies in children with juvenile idiopathic arthritis. J Rheumatol.

[43] Thastum M, Zachariae R, Schøler M, Bjerring P, Herlin T (1997). Cold pressor pain: comparing responses of juvenile arthritis patients and their parents. Scand J Rheumatol.

[44] Joharatnam N, McWilliams DF, Wilson D, Wheeler M, Pande I, Walsh DA (2015). A cross-sectional study of pain sensitivity, disease-activity assessment, mental health, and fibromyalgia status in rheumatoid arthritis. Arthritis Res Ther.

[45] Löfgren M, Opava CH, Demmelmaier I, Fridén C, Lundberg IE, Nordgren B (2018). Pain sensitivity at rest and during muscle contraction in persons with rheumatoid arthritis: a substudy within the Physical Activity in Rheumatoid Arthritis 2010 study. Arthritis Res Ther.

[46] Lee YC, Lu B, Edwards RR, Wasan AD, Nassikas NJ, Clauw DJ (2013). The role of sleep problems in central pain processing in rheumatoid arthritis. Arthritis Rheum.

[47] Vladimirova N, Jespersen A, Bartels EM, Christensen AW, Bliddal H, Danneskiold-Samsøe B (2015). Pain sensitisation in women with active rheumatoid arthritis: a comparative cross-sectional study. Arthritis.

[48] García-Fernández E, Godoy-Izquierdo D, Pérez-García M, Jiménez-Alonso J, López-Chicheri I, Godoy JF (2009). Differences in pressure-pain threshold between healthy women and patients with fibromyalgia syndrome, systemic lupus erythematosus, and rheumatoid arthritis. J Musculoskelet Pain.

[49] van Laarhoven AI, Kraaimaat FW, Wilder-Smith OH, van Riel PL, van de Kerkhof PC, Evers AW (2013). Sensitivity to itch and pain in patients with psoriasis and rheumatoid arthritis. Exp Dermatol.

[50] Barth S, Haas JP, Schlichtiger J, Molz J, Bisdorff B, Michels H (2016). Long-term health-related quality of life in German patients with juvenile idiopathic arthritis in comparison to German general population. PLoS One.

[51] Tollisen A, Selvaag AM, Aasland A, Lerdal A, Flato B (2019). Longitudinal health status from early disease to adulthood and associated prognostic factors in juvenile idiopathic arthritis. J Rheumatol.

[52] Schmelz M (2018). Quantitative sensory test correlates with neuropathy, not with pain. Pain..

