# Supplementary material for: Pain sensitivity in young adults with juvenile idiopathic arthritis: a quantitative sensory testing study
Source: Arthritis Res Ther. 2020 Nov 5;22:262. doi: 10.1186/s13075-020-02345-2 (PMC7643261; doi:10.1186/s13075-020-02345-2)
Supplement: Supplementary file 1 — Additional file 1: Supplementary Table S1. Thermal detection thresholds among juvenile idiopathic arthritis (JIA) and controls. [file 13075_2020_2345_MOESM1_ESM.pdf]

**Supplementary Table S1.** Thermal detection thresholds among juvenile idiopathic arthritis (JIA) and controls*

| | | **Cold detection threshold** Decrease below 32°C (95% CI) | | **Warm detection threshold** Increase above 32°C (95% CI) | |
|---|---|---|---|---|---|
| | n | Upper limb | Lower limb | Upper limb | Lower limb |
| Control | 109 | 1.2 (1.1, 1.3) | 2.0 (1.9, 2.2) | 2.1 (2.0, 2.2) | 4.1 (3.8, 4.4) |
| JIA (total group)[a] | 96 | 1.2 (1.1, 1.3) p =0.6 | 2.0 (1.8, 2.2) p =0.6 | 2.2 (2.1, 2.4) p =0.2 | 4.6 (4.2, 4.9) p =0.03 |
| JIA | | | | | |
| Remission off med.[b] | 43 | 1.3 (1.1, 1.4) p =0.3 | 2.1 (1.8, 2.4) p =0.8 | 2.1 (1.9, 2.3) p =1.0 | 4.7 (4.0, 5.3) p =0.09 |
| Inactive disease[b] | 20 | 1.2 (1.0, 1.3) p =0.9 | 1.9 (1.6, 2.2) p =0.4 | 2.3 (2.0, 2.7) p =0.2 | 4.4 (3.7, 5.1) p =0.5 |
| Active disease[b] | 33 | 1.1 (1.0, 1.3) p =0.7 | 1.9 (1.6, 2.2) p =0.5 | 2.3 (2.1, 2.5) p =0.09 | 4.6 (4.2, 5.0) p =0.04 |

*Multilevel modelling with re-transformed logarithm predicted values as difference in temperature from baseline (32°C) with 95% CI, adjusted for age and sex.

°C = degrees Celsius; CI = confidence interval; p = p-value comparing controls with JIA disease group (either total group or according to disease status).

[a]Separate multilevel model of the total JIA cohort.

[b]Disease status according to the definition by Wallace et al.; Remission off med. = Remission off medication for ≥12 months. Inactive disease = inactive disease on medication for less than 6 months or inactive disease off medication for less than 12 months or remission on medication (inactive disease on medication for more than 6 months). Active disease = flare or continuous active disease.
