# Supplementary material for: Pain sensitivity in young adults with juvenile idiopathic arthritis: a quantitative sensory testing study
Source: Arthritis Res Ther. 2020 Nov 5;22:262. doi: 10.1186/s13075-020-02345-2 (PMC7643261; doi:10.1186/s13075-020-02345-2)
Supplement: Supplementary file 2 — Additional file 2: Supplementary Table S2. Median thermal and pressure pain thresholds in juvenile idiopathic arthritis (JIA) and controls. [file 13075_2020_2345_MOESM2_ESM.pdf]

Supplementary Table S2. Median thermal and pressure pain thresholds in juvenile idiopathic arthritis (JIA) and controls

		**Cold pain threshold**[a]		**Heat pain threshold**		**Pressure pain threshold**	
		Absolute temperature °C		Absolute temperature °C		Kilopascal	
	n	Upper limb	Lower limb	Upper limb	Lower limb	Upper limb	Lower limb
Control	109	17.4 (10.0-24.2)	15.4 (5.9-23.0)	45.1 (41.6-47.2)	45.1 (43.2-46.8)	998 (918-1130)	721 (627-905)
JIA (total group)	96	19.1 (6.9-25.8)	16.6 (5.3-25.0)	44.7 (40.0-47.1)	44.8 (42.3-46.8)	890 (750-1014)	657 (579-793)
JIA							
Rem. off med.[b]	43	17.1 (5.8-24.6)	13.8 (5.1-25.0)	44.7 (39.3-47.3)	45.9 (42.0-47.9)	912 (785-1067)	702 (586-878)
Inactive disease[b]	20	20.5 (14.1-26.7)	23.0 (15.7-26.2)	44.4 (41.0-46.8)	43.3 (42.3-46.4)	818 (692-948)	617 (501-656)
Active disease[b]	33	18.1 (6.5-26.3)	13.8 (5.3-24.6)	44.9 (43.0-46.9)	44.9 (42.6-46.6)	910 (749-994)	697 (584-822)

Values are in median (interquartile range (IQR) 1th-3rd); °C = degrees Celsius; n = numbers.

[a]Due to the 5ºC floor temperature of the equipment, a substantial number of CPT responses reached this limit and were censored. The number (%) of censored CPT in JIA was 212 (28%) and in controls 198 (23%). Thus, median CPTs are underestimated compared to predicted values from the multilevel modelling in Table 3.

[b]Disease status according to the definition by Wallace et al.; Remission off med. = remission off medication for ≥12 months. Inactive disease = inactive disease on medication for less than 6 months or inactive disease off medication for less than 12 months or remission on medication (inactive disease on medication for more than 6 months). Active disease = flare or continuous active disease.
