# Supplementary material for: Pain sensitivity in young adults with juvenile idiopathic arthritis: a quantitative sensory testing study
Source: Arthritis Res Ther. 2020 Nov 5;22:262. doi: 10.1186/s13075-020-02345-2 (PMC7643261; doi:10.1186/s13075-020-02345-2)
Supplement: Supplementary file 3 — Additional file 3: Supplementary Table S3. Estimated differences in pain threshold per unit score in self-reported VAS pain. [file 13075_2020_2345_MOESM3_ESM.pdf]

Supplementary Table S3. Estimated differences in pain threshold per unit score in self-reported VAS pain

		Upper Limb			Lower Limb		
	n	Difference in pain threshold[a]	95% CI	p	Difference in pain threshold[a]	95% CI	p
**VAS current joint-related pain[b]**							
Cold pain threshold (CPT)							
All participants	204	0.19	-0.91 to 1.28	0.7	-0.28	-1.38 to 0.82	0.6
Controls	109	-0.79	-2.70 to 1.12	0.4	-1.02	-2.93 to 0.89	0.3
JIA	95	0.51	-0.98 to 2.00	0.5	-0.02	-1.52 to 1.48	1.0
Heat pain threshold (HPT)							
All participants	204	0.01	-0.33 to 0.36	0.9	-0.02	-0.29 to 0.26	0.9
Controls	109	-0.18	-0.74 to 0.38	0.5	-0.27	-0.69 to 0.15	0.2
JIA	95	0.16	-0.30 to 0.62	0.5	0.11	-0.22 to 0.44	0.5
Pressure pain threshold (PPT)							
All participants	204	-8.47	-26.41 to 9.46	0.4	-6.56	-23.60 to 10.48	0.5
Controls	109	3.78	-14.12 to 21.68	0.7	-12.18	-41.35 to 16.98	0.4
JIA	95	5.48	-17.34 to 28.30	0.6	2.07	-19.57 to 23.71	0.9
**VAS disease-related pain[c] (JIA)**							
Cold pain threshold (CPT)	96	0.03	-0.09 to 0.14	0.7	0.03	-0.09 to 0.15	0.6
Heat pain threshold (HPT)	96	0.03	-0.01 to 0.06	0.1	0.01	-0.01 to 0.04	0.3
Pressure pain threshold (PPT)	96	-0.05	-2.01 to 1.92	1.0	-0.40	-2.15 to 1.35	0.7

VAS = Visual analogue scale; n = numbers; CI = Confidence interval; p = p-value; JIA = Juvenile idiopathic arthritis.
[a]Regression coefficient representing difference in °C for CPT/HPT and kilopascal for PPT per unit change in VAS current joint-related pain/VAS disease-related pain.
[b]Self-reported current joint-related pain on the day of visit, measured with 10 cm continuous VAS (0 = no pain, 10 = unbearable pain).
[c]Self-reported disease-related pain during the last week measured with 21 numbered 0-10 VAS (0 = no pain, 10 = unbearable pain).
