# Supplementary material for: Pain sensitivity in young adults with juvenile idiopathic arthritis: a quantitative sensory testing study
Source: Arthritis Res Ther. 2020 Nov 5;22:262. doi: 10.1186/s13075-020-02345-2 (PMC7643261; doi:10.1186/s13075-020-02345-2)
Supplement: Supplementary file 4 — Additional file 4: Supplementary Table S4. Estimated differences in pain threshold: mild versus severe pain intensity / low versus high pain interference. [file 13075_2020_2345_MOESM4_ESM.pdf]

Supplementary Table S4. Estimated differences in pain threshold: mild *versus* severe pain intensity / low *versus* high pain interference

		Upper Limb			Lower Limb		
	n	Difference in pain threshold[a]	95% CI	p	Difference in pain threshold[a]	95% CI	p
**Pain intensity[b]**							
Cold pain threshold (CPT)							
All participants	205	2.00	-1.67 to 5.68	0.3	-1.51	-5.21 to 2.19	0.4
Controls	109	1.95	-3.14 to 7.04	0.5	-3.12	-8.23 to 1.99	0.2
JIA	96	1.47	-3.98 to 6.91	0.6	-0.59	-6.08 to 4.90	0.8
Heat pain threshold (HPT)							
All participants	205	0.18	-0.95 to 1.32	0.8	-0.02	-0.92 to 0.88	1.0
Controls	109	0.26	-1.20 to 1.72	0.7	0.50	-0.70 to 1.71	0.4
JIA	96	0.14	-1.52 to 1.79	0.9	-0.52	-1.74 to 0.71	0.4
Pressure pain threshold (PPT)							
All participants	205	-14.42	-80.75 to 51.91	0.7	-16.52	-76.44 to 43.41	0.6
Controls	109	21.98	-45.45 to 89.41	0.5	7.78	-84.39 to 99.95	0.9
JIA	96	-7.90	-103.22 to 87.41	0.9	-23.23	-98.07 to 51.60	0.5
**Pain interference[c]**							
Cold pain threshold (CPT)							
All participants	205	-2.92	-9.65 to 3.80	0.4	-4.76	-11.54 to 2.02	0.2
Controls	109	-1.42	-11.69 to 8.86	0.8	-5.61	-16.00 to 4.79	0.3
JIA	96	-3.78	-12.87 to 5.31	0.4	-4.01	-13.15 to 5.14	0.4
Heat pain threshold (HPT)							
All participants	205	-1.29	-3.56 to 0.98	0.3	-0.53	-2.68 to 1.62	0.6
Controls	109	-0.63	-3.48 to 2.22	0.7	1.04	-1.23 to 3.30	0.4
JIA	96	-1.46	-4.67 to 1.75	0.4	-1.31	-4.23 to 1.62	0.4
Pressure pain threshold (PPT)							
All participants	205	-46.98	-129.87 to 35.92	0.3	-70.01	-170.32 to 30.29	0.2
Controls	109	-29.01	-110.76 to 52.75	0.5	-44.80	-255.10 to 165.51	0.7
JIA	96	-17.36	-129.90 to 95.19	0.8	-68.61	-164.82 to 27.61	0.2

CI = Confidence interval; p = p-value; JIA = Juvenile idiopathic arthritis.

[a]Regression coefficient representing difference in °C for CPT/HPT and kilopascal for PPT between mild *versus* severe pain intensity and between low *versus* high pain interference.

[b]Self-reported body pain intensity during the last 4 weeks, measured with Short-form 36 Health Status Questionnaire (SF-36), 1-6 (1 = no pain, 6 = very severe pain), dichotomized into mild pain intensity (1-3) and severe pain intensity (4-6).

[c]Self-reported pain influence on daily activities during the last 4 weeks, measured with Short-form 36 Health Status Questionnaire (SF-36), 1-5 (1 = not at all, 5 = extremely), dichotomized into low pain interference (1-3) and high pain interference (5-6).
